# Rapid Surveillance for Vector Presence (RSVP): Development of a novel system for detecting *Aedes aegypti* and *Aedes albopictus*

**DOI:** 10.1371/journal.pntd.0005505

**Published:** 2017-03-24

**Authors:** Brian L. Montgomery, Martin A. Shivas, Sonja Hall-Mendelin, Jim Edwards, Nicholas A. Hamilton, Cassie C. Jansen, Jamie L. McMahon, David Warrilow, Andrew F. van den Hurk

**Affiliations:** 1 Metro South Public Health Unit, Queensland Health, Coopers Plains, Queensland, Australia; 2 Mosquito and Pest Management, Brisbane City Council, Fortitude Valley, Queensland, Australia; 3 Public Health Virology, Forensic and Scientific Services, Department of Health, Queensland Government, Coopers Plains, Queensland, Australia; 4 Rockhampton Public Health Unit, Queensland Health, Rockhampton, Queensland, Australia; 5 Institute for Molecular Bioscience, University of Queensland, St. Lucia, Queensland, Australia; 6 Metro North Public Health Unit, Queensland Health, Windsor, Queensland, Australia; Centers for Disease Control and Prevention, Puerto Rico, UNITED STATES

## Abstract

**Background:**

The globally important Zika, dengue and chikungunya viruses are primarily transmitted by the invasive mosquitoes, *Aedes aegypti* and *Aedes albopictus*. In Australia, there is an increasing risk that these species may invade highly urbanized regions and trigger outbreaks. We describe the development of a Rapid Surveillance for Vector Presence (RSVP) system to expedite presence- absence surveys for both species.

**Methodology/Principal findings:**

We developed a methodology that uses molecular assays to efficiently screen pooled ovitrap (egg trap) samples for traces of target species ribosomal RNA. Firstly, specific real-time reverse transcription-polymerase chain reaction (RT-PCR) assays were developed which detect a single *Ae*. *aegypti* or *Ae*. *albopictus* first instar larva in samples containing 4,999 and 999 non-target mosquitoes, respectively. ImageJ software was evaluated as an automated egg counting tool using ovitrap collections obtained from Brisbane, Australia. Qualitative assessment of ovistrips was required prior to automation because ImageJ did not differentiate between *Aedes* eggs and other objects or contaminants on 44.5% of ovistrips assessed, thus compromising the accuracy of egg counts. As a proof of concept, the RSVP was evaluated in Brisbane, Rockhampton and Goomeri, locations where *Ae*. *aegypti* is considered absent, present, and at the margin of its range, respectively. In Brisbane, *Ae*. *aegypti* was not detected in 25 pools formed from 477 ovitraps, comprising ≈ 54,300 eggs. In Rockhampton, *Ae*. *aegypti* was detected in 4/6 pools derived from 45 ovitraps, comprising ≈ 1,700 eggs. In Goomeri, *Ae*. *aegypti* was detected in 5/8 pools derived from 62 ovitraps, comprising ≈ 4,200 eggs.

**Conclusions/Significance:**

RSVP can rapidly detect nucleic acids from low numbers of target species within large samples of endemic species aggregated from multiple ovitraps. This screening capability facilitates deployment of ovitrap configurations of varying spatial scales, from a single residential block to entire suburbs or towns. RSVP is a powerful tool for surveillance of invasive *Aedes* spp., validation of species eradication and quality assurance for vector control operations implemented during disease outbreaks.

## Author summary

*Aedes* (*Stegomyia*) vectors of dengue, Zika and chikungunya viruses utilize artificial and natural containers as larval habitats. Adults do not usually disperse far (< 500 m) from these larval habitats in urban and peri-urban environments. Highly heterogeneous distributions raise significant logistic challenges to conduct informative surveillance. Public health imperatives require contemporaneous vector mosquito presence-absence data for highly urbanized regions that are both vulnerable to invasions and have frequent exposure to viremic travellers. We developed a promising tool to expedite presence-absence surveillance of *Aedes aegypti* and *Aedes albopictus* by integrating molecular diagnostics with ovitraps and automated egg quantification software. The high sensitivity of the molecular assays enabled samples from multiple ovitraps to be pooled and processed for each diagnostic test. This innovation resolves the considerable logistic constraints inherent in traditional ovitrap surveillance programs. Proof of concept was evaluated in field trials in Queensland geographies where *Ae*. *aegypti* is considered either absent, present or at the margin of its range (Brisbane, Rockhampton and Goomeri, respectively). *Aedes aegypti* was detected in Goomeri and Rockhampton and not detected in Brisbane. Further investigation is required to address the inaccuracy of automated egg counting software whenever contaminants are present. RSVP can accommodate varied ovitrap designs and deployment configurations, improves efficiency in laboratory and labor costs for high volumes of samples, and enables a rapid turnaround of results. The RSVP system can innovate surveillance programs for early-warning of invasion, eradication, and quality assurance for vector control in disease response contexts.

## Introduction

*Aedes aegypti* and *Aedes albopictus* are invasive mosquito species and global vectors of Zika (ZIKV) [1], dengue (DENVs) [2] and chikungunya (CHIKV) [3] viruses. Both species can coexist in the same ecological niche [4, 5] and share characteristics that are likely to make their detection difficult in the early stages of stochastic invasions, including heterogeneous distributions [6–8], limited dispersal capability of adults [9–13], and often low population densities [14–16]. Ovipositing females cement eggs inside a wide variety of water-bearing containers and these eggs can resist desiccation for several months [17, 18]. Transport to new regions or countries can occur via the shipment of immature stages in freight, such as used tires [19–21] and lucky bamboo [22, 23], or as adults sequestered in aircraft [24].

In Australia, quarantine authorities intercept both species at international seaports and airports [16, 25], with the frequency of detections increasing dramatically since 2012. However, there is a concurrent threat of range expansion from endemic Queensland populations. *Aedes aegypti* occurs throughout most of Queensland, although dengue outbreaks only occur in north Queensland [26–29]. *Aedes albopictus* has not colonized the Australian mainland following the rapid invasion of island communities of the Torres Strait, north Queensland [16, 30], largely due to suppression programs at transport hubs [31].

Importantly, both species are considered to be absent from southeast (SE) Queensland (population 3.4 million), where ≈ 70% of the Queensland population reside. This status is not based on systematic entomological baseline monitoring, but rather on the lack of local disease transmission following the importation of ZIKV, DENV and CHIKV in viremic travellers, coupled with negative results from *ad hoc* larval surveys and trapping of peridomestic mosquitoes. Southeast Queensland is considered to be receptive to invasion by both species. Indeed, *Ae*. *aegypti* was present in this region until the mid-1950s [29], whilst predictive models indicate the eastern seaboard of Australia could be colonized by *Ae*. *albopictus* [25, 32]. Receptivity is considered to be increasing [33, 34], partly due to the recent proliferation of water-storage containers, such as rainwater tanks [35]. In the future, ineffective mosquito-proofing of rainwater tanks or rainwater harvesting structures may increase the number of available larval habitats [36–38].

Contemporaneous baseline monitoring is essential to increase certainty that vector species are absent in geographies that are vulnerable to invasion [39] to minimize the risk of cryptic disease outbreaks. However, existing surveillance options for peridomestic mosquito species have operational weaknesses. Larval and pupal surveys are labor-intensive [40–42] and can be compromised by inaccessible premises or larval habitats, cryptic containers [43, 44], or timing a survey when mosquito abundance is low [16]. In terms of adult traps, some designs (e.g., Biogents BG-Sentinel trap) are highly sensitive [45] but require electricity and incur significant procurement, servicing, and maintenance costs [46, 47]. Novel adult traps (BG-Gravid *Aedes* Trap) are cheaper but less sensitive [48–50] and may be expensive to deploy in extensive arrays over a large spatial scale [6, 47, 51, 52]. Ovitraps provide a cheap and sensitive tool for presence-absence surveys of peridomestic species [17, 53, 54] but require an investment in time and laboratory resources to count eggs, rear and identify larvae [54, 55].

We report the development of a sensitive, easy-to-use, and cost-effective system, which we call Rapid Surveillance for Vector Presence (RSVP), to expedite presence-absence surveys of invasive *Aedes* species. The RSVP provides a powerful validation tool to confirm a species is absent at various spatial scales (property, suburban block, town or region), within an eradication program, and potentially as a quality assurance measure for vector control strategies implemented in response to ZIKV, DENV or CHIKV outbreaks.

## Methods

### Development and optimization of molecular assays to detect *Ae*. *aegypti* and *Ae*. *albopictus* in ovitrap samples

Eggs were hatched overnight by submerging ovistrips in 200 mL of Milli-Q water in rectangular 750 mL polyethylene containers, to which ≈ 10 mg of brain-heart powder was added to stimulate hatching. Ovistrips were removed and larvae extracted from the water via a vacuum filtration protocol. Specifically, the membrane and support pad of a MicroFunnel 300 filter funnel (Pall Life Sciences, Ann Arbor, MI, USA) was replaced with an FTA card (GE Healthcare Life Sciences, PA, USA) trimmed to fit the base of the funnel. The filter funnel was then placed on top of a vacuum flask. The water containing the larvae was gently agitated before it was poured into the cylinder of the filter funnel and a vacuum applied. The filter funnel and hatching container were rinsed with Milli-Q water until there were no larvae visible. The cards were then removed from the base of the funnel and air-dried overnight at room temperature.

The protocol of Hall-Mendelin et al. [56] was used to prepare the FTA cards and dried larvae for nucleic acid extraction. Briefly, the cards were cut into 4–5 strips and placed in a 5 mL vial containing 1 mL of Milli-Q water. Vials were placed on wet ice and vortexed every 5 min for 15 sec for a total of 20 min. The cards were then placed in a 5 mL syringe from which the plunger had been removed. The plunger was used to squeeze the liquid from the cards into a 2 mL vial. Nucleic acids were extracted from 140 μL of the eluate using the Bio Robot Universal System (Qiagen, Hilden, Germany) and the QIAamp Virus BioRobot MDx Kit (Qiagen, Clifton Hill, Australia) according to the manufacturer’s instructions.

Real-time TaqMan reverse transcription-polymerase chain reaction (RT-PCR) assays were used to detect target *Ae*. *aegypti* and *Ae*. *albopictus* against a background of endemic species. The *Ae*. *aegypti* ribosomal RNA was detected using real-time TaqMan RT-PCR with forward GCAGTCAGATGTTTGATACCGC and reverse GGTTGACGTATTATCAGGTCACACTA primers at 500 nM, and probe FAM-TGGGCGCCTCGGTGTCCCG-TAMRA at 300 nM. The *Ae*. *albopictus* ribosomal RNA was detected using forward CCGACAAGGCAATATGTC and reverse ACGCGTACGGACATTG primers at 300 nM, and probe FAM-TTCCCTCCGATCAGCGAACTC-TAMRA at 200 nM. The cycling conditions consisted of one cycle at 50°C for 5 min, one cycle at 95°C for 2 min, and 40 cycles at 95°C for 3 sec and 60°C for 30 sec. A positive result, indicating the presence of target species RNA, corresponded to cycle threshold values of ≤ 40 cycles. Reaction controls always included a no-template and synthetically generated template samples. The specificity of the 2 TaqMan RT-PCR assays was tested using 4^th^ instar larval samples of *Ae*. *aegypti* and *Ae*. *albopictus*, as well as other peridomestic species, including *Aedes katherinensis*, *Aedes notoscriptus*, *Aedes palmarum*, *Aedes scutellaris*, *Aedes tremulus* and *Culex quinquefasciatus*.

Pools of varying sizes were produced to test the sensitivity of the TaqMan RT-PCR assays in detecting *Ae*. *aegypti* and *Ae*. *albopictus*. The *Ae*. *aegypti* eggs used to optimize the sensitivity of the assay were obtained from the F_2-4_ generations of a colony originating from Townsville, Australia, while F_o_ *Ae*. *notoscriptus* were collected in ovitraps deployed in Brisbane, Australia. The *Ae*. *albopictus* were from a colony established from eggs collected from Hammond Island (Torres Strait, Australia) and had been in colony for < 10 generations. Following hatching as described above, single 1^st^ instar *Ae*. *aegypti* or *Ae*. *albopictus* larvae were added to batches of 1^st^ instar *Ae*. *notoscriptus* or *Ae*. *aegypti* larvae, respectively, to produce pool sizes of 10, 100, 1,000 and 5,000 larvae. In addition, a pool comprised of a single *Ae*. *aegypti* or *Ae*. *albopictus* 1^st^ instar larvae, and pools containing only *Ae*. *notoscriptus* or *Ae*. *aegypti* 1^st^ instar larvae were produced. The pools were processed using the vacuum filtration method described above and *Ae*. *aegypti* and *Ae*. *albopictus* detected using the species*-*specific TaqMan RT-PCR assays.

### Field validation of the RSVP as a tool for detection of *Ae*. *aegypti*

#### Study sites

Three sites were chosen to represent the trichotomy of *Ae*. *aegypti* distribution in Queensland. Brisbane (27° 28’15.6”S, 153°1’24.4”E) is the state capital (> 1 million population; > 380,000 private dwellings) [57] within SE Queensland with no recent detections of *Ae*. *aegypti*. The city provided a large geographical area (> 1,367 km^2^) to test RSVP in a species absence context. Rockhampton (-23°22’49”S, 150°30’21”E) is a small regional city 636 km NW of Brisbane (80,000 population; > 32,000 private dwellings). *Aedes aegypti* is locally abundant and therefore provided a suitable site for field validation of species presence. Goomeri is a small town (< 500 population, > 200 private dwellings), 235 km NW of Brisbane and represents the ill-defined margin of *Ae*. *aegypti* distribution nearest SE Queensland. *Aedes aegypti* has previously been detected during infrequent larval surveys.

#### Trapping strategy

Large ovitraps were assembled to maximize visual and olfactory cues [58, 59]. Black plastic buckets (9.3L) were provided with an overflow hole and exposed to the outdoor environment for 2 weeks prior to deployment, to remove volatile chemicals that may repel ovipositing females. An ‘ovistrip’ [60] (380 x 50 mm) made from white Tork Cleaning Cloth (SCA, Stockholm, Sweden) was affixed to the inside wall of each bucket with a fold-back clip. A pellet of compacted alfalfa was added to 3-L of tap water to produce an organic infusion [61]. Black plastic mesh covers (20 mm aperture) were fitted to prevent access by children or domestic animals.

**Fig 1:**
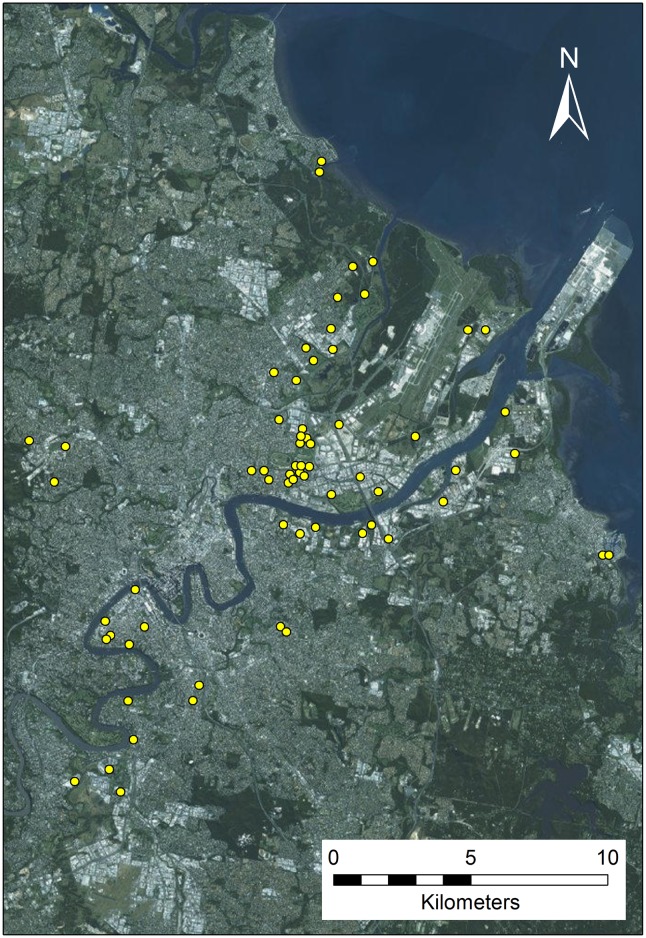
Spatial distribution of RSVP ovitraps (*n* = 477) in Brisbane deployed at commercial and residential dwellings (29 January- 9 July 2015). The map layer was created using ArcGIS Online (https://www.arcgis.com/features/index.html).

Ovitrap sites included well-shaded urban yards, premises near imported dengue cases, commercial precincts and transport hubs (Fig 1). Ovitraps were deployed with occupier consent in outdoor sites that were adjacent to premises and protected from rain and wind [62]. Brisbane ovitraps were primarily deployed in a routine monitoring context from January to July 2015 and were collected after 2 weeks. Rockhampton ovitraps were deployed in May and June 2015, and were collected after 7 days. Goomeri ovitraps were deployed in February and March 2016 for periods ranging from 11–15 days. Ovistrips were transferred to the local office in individual sealable bags and dried overnight at room temperature. Brisbane ovistrips were pooled each fortnight, whilst additional *ad hoc* pools were submitted from suburbs where an imported dengue case was notified. Rockhampton ovistrips were pooled according to geographical clusters, or the suburb where an imported case of dengue was notified. Goomeri ovistrips were pooled per each trapping event. All ovistrips were forwarded to Forensic and Scientific Services, Department of Health, Queensland Government, Brisbane, for molecular identification.

### Egg quantification using image analyses

**Fig 2:**
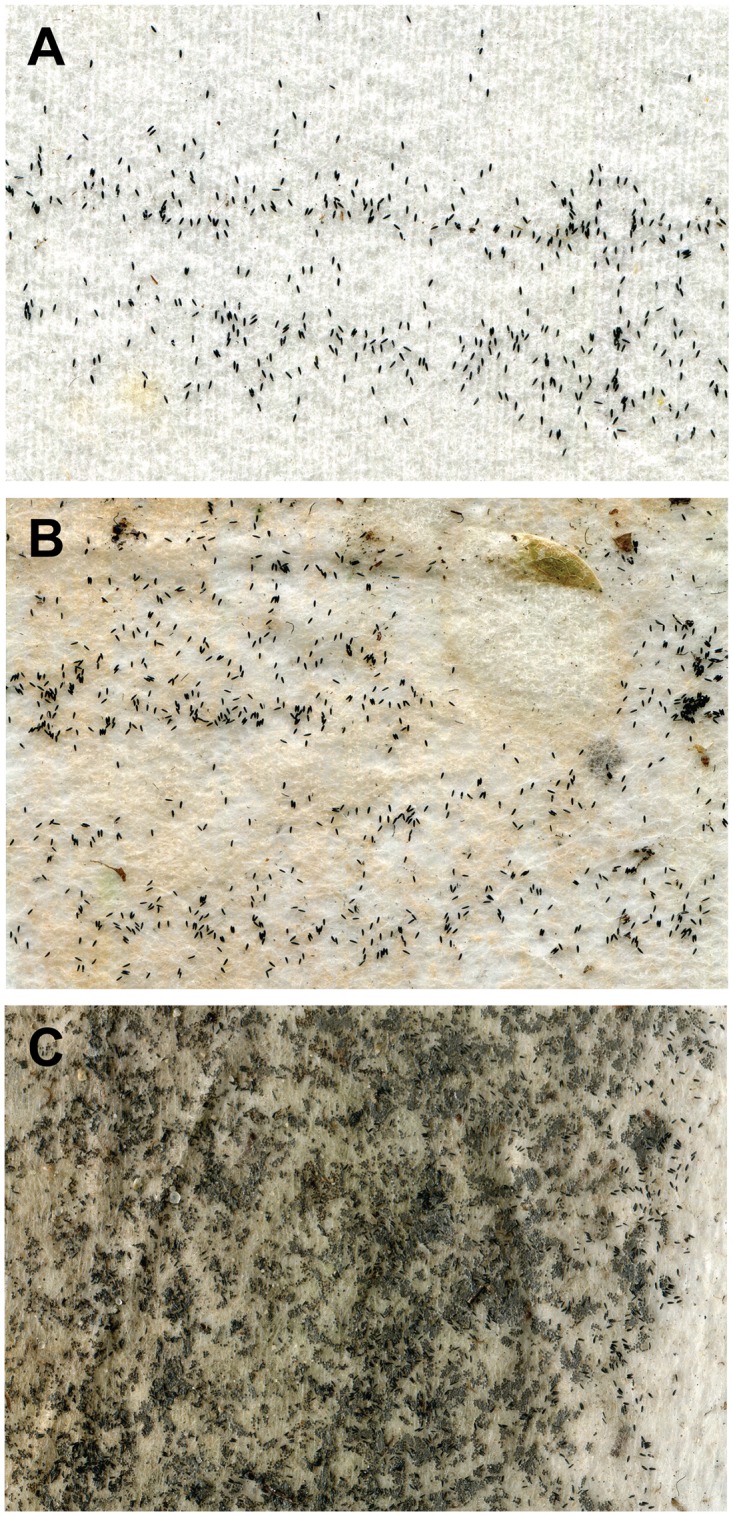
Examples of ovistrip condition (*n* = 193) for submission to quantify the number of eggs by ImageJ analyses. (A) Image suitable for immediate analysis; (B) image requires manipulation for image analysis; and (C) image unsuitable for analysis.

We assessed ImageJ software [63] as a platform to count egg numbers on ovistrips *in situ* from Brisbane ovitraps deployed during the 2013–14 summer. Using a stereo microscope, each ovistrip was inspected, eggs counted and relative contamination assessed to ascertain whether the ovistrip was of sufficient quality to be analyzed by ImageJ (Fig 2). Quality of the ovistrips was especially important, as ImageJ cannot differentiate *Aedes* eggs from other objects or contaminants, such as debris, leaves, insect cadavers and/or fungal growth, on the ovistrip. This could potentially produce an inaccurate count of the eggs on the ovistrips. Consequently, an arbitrary grade for each ovistrip was assigned to determine suitability for ImageJ analysis and comprised suitable (Fig 2A), suitable with image manipulation (Fig 2B) and unsuitable (Fig 2C).

An image of each ovistrip was then captured using a standard flatbed scanner (Epson Perfection V370 Photo, Suwa, Nagano, Japan). Images were saved in .jpg format under standardized conditions of ovistrip presentation, background, debris removal and a fixed resolution (1200 dpi). We created a macro to analyze each image individually within a directory of imagery. The image was initially split into red, green and blue colour channels. In the red channel, eggs appeared as dark regions on a light background. Accurate selection and measurement of dark regions required the image to be smoothed with a median filter (radius 2 pixels) and inverted (eggs appear as light regions on a dark background). A threshold was then applied (intensity 140) to binarize the image so that egg regions were rendered white (intensity 255) on a black background (intensity 0).

ImageJ produces two estimates of egg number; the number of distinct/disjoint eggs or egg regions (the unit method), and the total area (in pixels) of egg regions (the area method). For the latter method, an average value of 168 pixels per *Ae*. *notoscriptus* egg (*n* = 25) was calculated.

The script was automated upon selection of a directory and dual results (a spread sheet containing quantification for each image and binary images showing the regions selected by the macro as being egg-covered) placed in a subdirectory. The binary images provided a quality control mechanism to check that the macro functions correctly and egg regions are located accurately.

## Results

### Sensitivity of the novel real-time TaqMan RT-PCR assays for detection of *Ae*. and *Ae*. *albopictus*

The *Ae*. *aegypti* TaqMan RT-PCR assay was able to detect single 1^st^ instar larvae in all pools that contained increasing numbers of *Ae*. *notoscriptus* up to a pool size of at least 5,000 (Table 1). The *Ae*. *albopictus* assay was not as sensitive, with the limit of detection being a single 1^st^ instar larva in a pool of 1,000 *Ae*. *aegypti*.

**Table 1:** Identification of single 1st instar *Ae*. *aegypti* or *Ae*. *albopictus* larvae in pools of 1st instar *Ae*. *notoscriptus* or *Ae*. *aegypti* larvae, respectively using the TaqMan RT-PCR assays.

	*Aedes aegypti*		*Aedes albopictus*	
Species ratio^a^	*n*^b^	Mean ± SD C_t_ value	*n*	Mean ± SD C_t_ value
1:0	5/5	12.6 ± 0.5	5/5	21.6 ± 0.7
1:9	5/5	12.8 ± 1.7	5/5	19.9 ± 0.6
1:99	5/5	13.9 ± 2.2	5/5	20.9 ± 0.6
1:999	5/5	27.6 ± 4.5	5/5	22.4 ± 1.1
1:4,999	2/2	27.6 ± 3.3	0/2	> 40.0

^a^Ratio of individual *Ae*. *aegypti* or *Ae*. *albopictus* to *Ae*. *notoscriptus* or *Ae*. *aegypti*, respectively, in pools of first instar larvae.

^b^n = number of pools detected/number tested; A sample was detected if the cycle threshold (C_t_) value was < 40.0 cycles. Ct values > 40.0 were considered to be not detected.

### Field validation of the RSVP system

A total of 477 ovitraps were deployed in Brisbane of which 400 (83.9%) collected >75,000 *Aedes* spp. eggs. The processing of 25 pools, representing an estimated ≈ 54,400 eggs, using the *Ae*. *aegypti* TaqMan assay did not detect *Ae*. *aegypti*.

A total of 45 ovitraps were deployed in Rockhampton, of which 18 (40.0%) collected an estimated total of ≈ 1,700 *Aedes* spp. eggs. *Aedes aegypti* was detected in 4 of 6 pools.

A total of 62 ovitraps were deployed in Goomeri, of which 32 (51.6%) collected an estimated ≈ 4,200 eggs. *Aedes aegypti* was detected in 5 of 8 pools.

### Utility of image analysis for quantifying eggs on ovistrips

Due to an absence of visual contaminants, the eggs on 107 of 193 (55.4%) of ovistrips were able to be accurately quantified using ImageJ. Of the remainder, accretion of contaminants rendered 41 of 193 (21.2%) of ovistrips unsuitable for analysis. The eggs on 45 of 193 (23.3%) ovistrips could be quantified with manipulation, such as removing debris or digitally enhancing the image.

## Discussion

The RSVP system is a powerful and flexible tool for presence-absence surveillance of invasive *Aedes* (*Stegomyia*) mosquito species. RSVP utilizes sensitive molecular diagnostics to resolve logistical and time constraints typically associated with ovitrap surveillance. Molecular diagnoses take approx. 48 h, including hatching of eggs, nucleic acid extraction and real-time RT-PCR. This represents an appreciable time saving, considering that 1,000s of larvae from multiple ovitraps can be processed concurrently. In comparison, ovitraps require approx. 7–10 days to rear cohorts to 4^th^ instar larvae or adults before each specimen is identified by the microscopic examination of key morphological characters. Molecular analyses mitigates the risk of human error in detecting a small number of specimens of an invasive species that are obscured by many 1,000s of larvae from endemic species [64]. Hatching eggs to 1^st^ instar larvae prior to RT-PCR analysis removes requirements for a specific ovitrap format (design, size, material), ovistrip size [65] or substrate (e.g., cloth, paper, germination paper, wood). Shipment of large numbers of egg samples by postal service is also potentially more logistically viable than couriering water samples for newly developed environmental DNA analysis [66].

When viewed in isolation, this application of molecular diagnostics may seem expensive, with a single test costing approx. AU$50.00. However, compared to morphological identification, the saving in time and labor to perform one molecular diagnosis for 10 pooled samples (or AU$5.00 per ovitrap) more than offsets the cost of performing the molecular assays. Furthermore, the cost to perform each test will decrease as the number of cards tested increases. Molecular diagnostics can detect multiple target species (e.g. *Ae*. *albopictus* and *Ae*. *aegypti*) once the nucleic acids have been extracted, with only a marginal cost increase to include an additional species. Other real-time TaqMan assays can be developed to identify other invasive species, such as *Aedes japonicus* and *Aedes koreicus*, which have recently colonized North America and Europe, respectively [67].

Estimates of egg abundance on each ovistrip will guide the aggregation of ovistrips into pool sizes that are below the TaqMan RT-PCR assay’s threshold of detection, and enable comparisons between trap sites or to generate spatial ‘heat maps’ [7]. Egg counting software has the potential to remove the need for manual counting and improve the accuracy and consistency of subjective rapid visual estimates [68]. We demonstrated that systems developed to count eggs harvested under laboratory conditions [69, 70] may not be suitable for rapid analyses of field collections due to greater exposure of ovistrips to contaminants. It should be noted that eggs on any contaminated ovistrips would remain viable for molecular analyses. Pre-trial testing in Brisbane determined that a 2 week ovitrap deployment minimized the accretion of contaminants, such as plant debris and/or bacterial and fungal growth. Whilst the relatively high number of contaminated ovistrips limited its accuracy for automated egg counting in our trials, ImageJ or other image processing software warrants further investigation.

The spatial scale of an RSVP ovitrap network can be adjusted to service early-warning, eradication or quality assurance programs. RSVP was trialled by inexperienced field staff in locations where *Ae*. *aegypti* is present (Rockhampton), absent (Brisbane) and near the margin of its geographic range (Goomeri). The simplicity, operational flexibility and the rapidity of diagnosis that RSVP provided was well received by these staff. The RSVP system is currently being translated into an expanded local government program in southeast Queensland. Furthermore, the RSVP is also providing a diagnostic platform for a pilot citizen-science project in southeast Queensland. This pilot will emulate citizen-science programs that monitor invasive mosquitoes in Europe [71] and North America (Invasive Mosquito Project), and may complement community-release programs for novel biological control strategies, such as *Wolbachia*-based programs [72–74].

RSVP may provide a powerful quality assurance tool [75] for ZIKV, DENV or CHIKV responses, either through rapid analyses of eggs from lethal ovitraps [60, 76] or non-lethal ovitraps. Vector presence-absence data within small spatial scales (e.g. residential block) [27, 77, 78] may facilitate risk-based assessment for persistence of local transmission and direct subsequent control operations toward blocks that are infested. The RSVP has potential to be integrated with the roll-out of *Wolbachia*-based programs. For example, ovitraps were used to assess the rate of invasion by *Ae*. *aegypti* infected with the endosymbiont *Wolbachia pipientis* in open field releases in north Queensland [78]. RSVP may also provide a tool to facilitate the measurement of the heterogeneity of *Ae*. *aegypti* in the assessment of candidate sites before releases [79], or aid the assessment of *Wolbachia* invasion during and after releases for interventions aimed either at the reduction of vector populations [80–82] or for control of ZIKV [83, 84], DENV [77, 85, 86] and CHIKV [87, 88] outbreaks.
